# Application of Molecularly Imprinted Polymers in the Analysis of Explosives

**DOI:** 10.3390/polym17101410

**Published:** 2025-05-20

**Authors:** Chenjie Wei, Lin Feng, Xianhe Deng, Yajun Li, Hongcheng Mei, Hongling Guo, Jun Zhu, Can Hu

**Affiliations:** 1 School of Investigation, Peoples’ Public Security University of China, Beijing 100038, China; 18903560186@163.com (C.W.); pooh_feng@126.com (L.F.); 2 Insititute of Forensic Science, Ministry of Public Security, Beijing 100038, China; lihua0451@126.com (X.D.); lyjnumber1@126.com (Y.L.); meihongcheng@163.com (H.M.); guohongling1234@163.com (H.G.)

**Keywords:** molecularly imprinted polymers, explosives, sample pretreatment, sensors

## Abstract

The detection of explosives is highly important for the investigation of explosion cases and public safety management. However, the detection of trace explosive residues in complex matrices remains a major challenge. Molecularly imprinted polymers (MIPs), which mimic the antigen–antibody recognition mechanism, can selectively recognize and bind target explosive molecules. They offer advantages such as high efficiency, specificity, renewability, and ease of preparation, and they have shown significant potential for the efficient extraction and highly sensitive detection of trace explosive residues in complex matrices. This review comprehensively discusses the applications of MIPs in the analysis of explosives; systematically summarizes the preparation methods; and evaluates their performance in detecting nitroaromatic explosives, nitrate esters, nitroamine explosives, and peroxide explosives. Finally, this review explores the future potential of emerging technologies in enhancing the MIP-based analysis of explosives. The aim is to support the further application of MIPs in the investigation of explosion cases and safety management, providing more effective technical solutions for public safety.

## 1. Introduction

Serious explosion incidents can cause enormous loss of life and property and have significant social impacts [1,2,3]. Explosions may result from terrorist attacks, ordinary criminal offenses, or accidents caused by management negligence. There is an urgent need for rapid investigation methods to effectively combat crime and safeguard people’s lives and property. The detection of explosives is crucial for the investigation of explosion cases. Currently, a series of methods for analyzing explosives has been established [4,5,6]. However, the matrices at explosion scenes are complex and often exhibit severe interference, while most explosive residues exist at low concentrations, making them difficult to extract. Therefore, the efficient extraction and highly sensitive detection of trace explosive residues in complex matrices have remained research hotspots.

**Figure 1:**
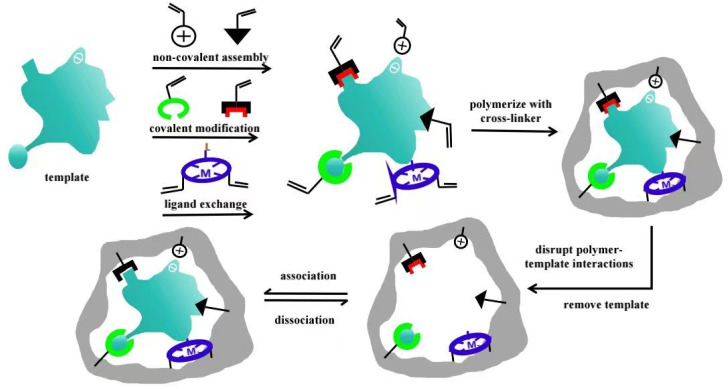
Schematic of molecularly imprinted polymers [9].

Molecular imprinting technology mimics the antigen–antibody recognition mechanism in biological systems. These polymers can be custom designed and synthesized for specific target molecules. The resulting polymers can specifically recognize and selectively adsorb target template molecules and their structural analogs, enabling efficient recognition and separation [7]. Molecularly imprinted polymers (MIPs) are formed via the cross-linking polymerization of functional monomers and template molecules in the presence of an initiator. The template molecules are subsequently removed from the polymer via specific methods, resulting in the formation of recognition sites that are complementary to the template in both spatial configuration and functional group distribution (Figure 1). MIPs demonstrate high efficiency, remarkable specificity, renewability, and ease of preparation. Thanks to their diverse and excellent properties [8], MIPs show great promise in a wide range of applications, including solid-phase extraction (SPE) and sensor technologies.

MIPs also have extensive applications in the analysis of explosives. Through customized design and synthesis, MIPs can be tailored to exhibit high selectivity and sensitivity toward specific explosive molecules. These characteristics enable MIPs to offer robust technical support for the accurate identification and quantification of explosive components. Researchers have thoroughly investigated various preparation methods for MIPs used in explosive analysis, along with performance evaluation criteria and applications in areas such as sample pretreatment and sensor-based detection. Significant progress has been made in the separation, enrichment, and purification of explosive components [10,11,12].

Many scholars have reviewed the research progress in this field [13,14,15,16]. However, few papers have systematically summarized the preparation methods for MIPs and their detection performance for different types of explosives. Therefore, this article provides a comprehensive review of the applications of MIPs in the analysis of various explosives, including nitroaromatic explosives, nitrate esters, nitroamine explosives, and peroxide explosives, in recent years. It also offers an in-depth discussion of the advantages and limitations of these applications. Moreover, this review examines the potential of cutting-edge technologies in enhancing MIP-based explosive analysis and anticipates future trends in this field. The ultimate aim is to promote broader applications of MIPs in explosive detection and contribute more effectively to public safety.

## 2. Preparation Methods for Molecularly Imprinted Polymers Used in the Analysis of Explosives

### 2.1. Preparation Techniques

#### 2.1.1. The Entrapment Method

The entrapment method is the most commonly used traditional technique in the preparation of MIPs. As the name implies, this method refers to a preparation technique in which most of the imprinted sites of the prepared polymer are cross-linked and entrapped inside the product, with limited surface site distribution. Common entrapment methods include bulk polymerization, precipitation polymerization, emulsion polymerization, and suspension polymerization.

Bulk polymerization was the most commonly used method for preparing MIPs in the early days. This method offers several advantages. The polymerization process is straightforward, the reaction conditions are mild, and there are minimal interfering factors. However, the posttreatment process of the polymer prepared by this method is complex, time-consuming, and labor intensive. Moreover, operations such as grinding cause a certain degree of damage to the imprinted cavities and result in material waste. In addition, the obtained polymer exists in block form, featuring irregular shapes and large particle sizes. This situation leads to significant resistance during the elution and adsorption of the polymer. As a result, the utilization rate of the binding sites is low. Alexander et al. [17] reported that imprinted polymer particles prepared by bulk polymerization have irregular shapes and sizes and poor dispersibility, which restricts their application in high-performance liquid chromatography (HPLC), capillary electrochromatography (CEC), and SPE.

Compared with bulk polymerization, precipitation polymerization is initiated in a polymerization system with excess solvent. Precipitation polymerization was first reported by Ye et al. [18,19]. The precipitation polymerization method has obvious advantages. There is no need to add stabilizers or surfactants to the preparation system. The prepared polymer microspheres have a clean surface, regular particle shapes, and good imprinting effects, and the obtained particles do not require complex posttreatment processes such as grinding [20]. Lu et al. [21] employed the precipitation polymerization method to prepare MIPs and corresponding nonimprinted polymers (NIPs) using trinitrotoluene (TNT) or 2,4-dinitrotoluene (2,4-DNT) as templates. These authors subsequently integrated these polymers with ion mobility spectrometry (IMS) for the efficient and sensitive detection of nitrobenzene compounds in surface water. Their work thereby presented a novel technical approach for environmental monitoring. Stringer et al. [22] prepared MIP microparticles by precipitation polymerization, using water or chloroform as the polymerization solvent. Chloroform was proven to be the best solvent for the molecular imprinting of nitroaromatic explosive compounds.

Emulsion polymerization is a powerful tool for synthesizing particles with sizes as small as a few nanometers. The synthesized nano-MIPs have uniform and accessible binding sites. However, many chemical reagents, including surfactants, buffers, and stabilizers, must be added during the synthesis process. These components usually require complex washing procedures to remove them after synthesis, which not only increases the operation difficulty but also reduces the purity of the material [23]. Xu et al. [24] prepared quantum dots (QDs) modified with MIPs via the reverse microemulsion method, Stober method, and seed growth method. Moreover, NIP-modified QDs were prepared as controls. A novel fluorescence sensor, dummy MIP (DMIP)@QDs, based on the fluorescence quenching mechanism induced by electron transfer, was developed for the detection of TNT. This development holds great significance in the field of environmental monitoring.

Suspension polymerization is the most commonly used and simplest method for preparing polymer microspheres. Compared with bulk polymers produced by bulk polymerization, this method results in a larger specific surface area. However, the presence of strongly polar solvents likely destroys the noncovalent interactions between monomers and template molecules, resulting in the poor recognition performance of the prepared polymers. To address this issue, Mayes et al. [25] initially employed perfluorohydrocarbons as the dispersion phase in precipitation polymerization. They successfully obtained stable polymerized droplets and ultimately fabricated polymer microspheres with outstanding performance. However, the method of Mayes et al. requires the addition of fluorinated surfactants, and perfluorohydrocarbons are relatively expensive and not suitable for large-scale use. With the expansion of the application fields of MIPs, the seed-swelling suspension polymerization method, with which the preparation and adsorption of MIPs can be completed in aqueous systems, has attracted the attention of researchers [26]. Cheng Guoxiang et al. [27] used tyrosine as the imprinted molecule, methacrylic acid (MAA) as the functional monomer, and trimethylolpropane trimethacrylate (TRIM) as the cross-linker to prepare MIP microspheres in an aqueous solution. The microspheres synthesized via this method exhibited excellent monodispersity, with micropores distributed on their surfaces. Moreover, they demonstrated remarkable specific adsorption properties. The method of Cheng Guoxiang et al. not only solves the issue of interference caused by high polarity and water but also results in smaller polymer microspheres, effectively avoiding the problems of grinding and sieving.

#### 2.1.2. New Techniques

With the rapid development of science and technology, new preparation techniques for MIPs have continuously emerged, bringing new opportunities and challenges to the field of analysis of explosives. Researchers have conducted in-depth explorations of these new techniques, which not only improve the preparation efficiency and quality of MIPs but also expand their application scope in the analysis of explosives.

Surface molecular imprinting technology is a technique for preparing MIPs on the surface of a carrier. It was first reported by Mosbach et al. [28]. The MIPs prepared in this way can have binding sites distributed on the surface of the polymer. On the one hand, this approach enables the more thorough removal of template molecules from the synthesized MIPs. As a result, it mitigates the influence of residual template molecules on the recognition capacity of MIPs and enhances the template removal efficiency. On the other hand, it enables MIPs to precisely capture target explosive molecules within complex matrices. This makes the target molecules more readily accessible, significantly shortens the adsorption equilibrium time, and enhances the binding efficiency between MIPs and the target explosive molecules. In the field explosives’ analysis, surface molecular imprinting techniques mainly involve the sacrificial carrier method, electropolymerization method, and carrier method [29,30,31].

Xiang Weizhong [32] used thin-layer chromatography silica gel as a sacrificial carrier. After the imprinting process was complete, the silica gel carrier was washed away with hydrofluoric acid. According to the study of Xiang Weizhong, the prepared polymer particles were uniform and regular, and their specific adsorption ability was significantly enhanced compared with that of the polymers prepared by the traditional entrapment method.

Guo et al. [31] used TNT as the template molecule and formed MIP microporous metal–organic frameworks (MMOFs) in situ, on the surface of a gold electrode, through electropolymerization of 4-aminothiophenol-functionalized gold nanoparticles. The modified electrode showed sensitive recognition characteristics for TNT, with a linear response within the range of 4.4 fmol/L to 44 nmol/L, a detection limit of 0.04 fmol/L, and good reproducibility for the detection of TNT. It can be used to detect TNT in complex matrices such as water from faucets and natural water bodies. The study shows that many significant advantages of surface molecular imprinting technology make the application of MIPs in the analysis of explosives more flexible and efficient.

Shunying et al. [33] prepared surface MIPs for arsenate detection through computer-aided design and achieved highly selective adsorption and detection of arsenate. This research not only demonstrates the powerful ability of surface molecular imprinting technology to realize the recognition of specific target molecules but also provides a useful reference for the design and preparation of surface MIPs for analysis of explosives. By drawing on similar research methods and ideas, more surface MIPs for specific explosive molecules can be designed to further explore the application potential of surface molecular imprinting technology in the detection, separation, and enrichment of explosives, providing more accurate and efficient analytical tools for analysis of explosives.

In situ polymerization is an innovative method for directly synthesizing MIPs on a specific matrix or carrier. Compared with traditional methods, in situ polymerization reduces the number of grinding and packing steps and improves the preparation efficiency. Zhao Chen et al. [34] harnessed this technique and integrated it with fluorescence molecular imprinting to enable the sensitive and rapid detection of the explosive molecule TNT. Their method achieved a detection limit of 0.01 mg/mL, comprehensively demonstrating the application value of in situ polymerization in the analysis of explosives. In addition, the MIPs prepared by in situ polymerization not only have a high degree of structural regularity but also exhibit excellent stability.

Yang Zhijian [35] polymerized a functional monomer on the surface of a nitroamine explosive in situ, enabling the nitroamine explosive to be encapsulated inside the polymer. Experiments indicated that the polymer-coated explosive produced by this method had excellent thermal stability. Moreover, in impact sensitivity and impact shock tests, it demonstrated good stability and a certain degree of resistance to impact energy. In situ polymerization also has the advantages of a simple preparation process, easy control, and large-scale production. These advantages make in situ polymerization technology more widely and practically applicable in the analysis of explosives. Moreover, in situ polymerization technology has also demonstrated great potential in the preparation of magnetic MIPs.

Flora et al. [36] prepared magnetic dual-template MIPs by using in situ polymerization technology and applied them to the detection of triazine pesticides. This study fully demonstrated the powerful ability and application prospects of in situ polymerization technology in the preparation of magnetic MIPs. We can build upon this technical concept and fabricate magnetic MIPs by incorporating magnetic nanoparticles as carriers. This will enable the magnetic separation and enrichment of explosive molecules. These magnetic MIPs will not only possess a high level of selectivity and sensitivity but can also be used to streamline subsequent analyses and processing procedures. As a result, they will significantly enhance the overall analysis efficiency.

In addition to surface molecular imprinting technology and in situ polymerization technology, molecularly imprinted hollow spheres (MIHSs), which are innovative polymers, also provide new ideas and methods for the analysis of explosives. MIHSs are a type of MIP with a hollow structure. Their internal cavities can serve as binding sites for explosive molecules, improving the selectivity and adsorption capacity of MIPs [37].

R. Pohle et al. [38] detected explosives based on work-function-readout MIPs, demonstrating the advantages of the MIHSs’ structure in selectively adsorbing and enriching target molecules. Drawing on the method proposed by Chen et al. [39], Wang Jian [40] optimized the functional monomers, the cross-linkers, and their ratios to the template in MIPs. Through the sacrificial carrier method, Wang Jian prepared MIHSs of hexanitrohexaazaisowurtzitane (CL-20). He further devised an SPE protocol. Through the optimization of the sample loading, washing, and elution solvents, this protocol allows the simultaneous SPE of multiple explosives from complex samples contaminated with engine oil and vacuum pump oil. As a result, the treated samples can be directly analyzed for multiple explosives using an HPLC diode array detector (HPLC-DAD).

In the analysis of explosives, we can further draw on this technology to prepare MIHSs with a high degree of selectivity and a high adsorption capacity for specific explosive molecules. Such a novel polymer would not only offer benefits such as a large specific surface area and a rapid mass transfer rate but also has the potential to be integrated with other techniques, such as magnetic separation and fluorescence detection. This integration would enable the efficient separation and detection of explosive molecules [41]. Through the optimization of the preparation conditions and parameters for MIHSs, including the selection of suitable monomers, cross-linkers, porogens, and other factors, their adsorption performance and selectivity can be further enhanced. Future research can explore the application of MIHSs in the analysis of explosives more deeply, providing more accurate and efficient analytical tools for the detection of explosives.

### 2.2. Preparation Methods for Molecularly Imprinted Polymers for the Detection of Different Explosives

The polymerization process in the preparation of MIPs involves multiple experimental parameters, including template molecules, functional monomers, cross-linkers, solvents (porogens), initiators, and initiation methods. These experimental parameters can affect the morphology, chemical properties, and recognition performance of molecularly imprinted materials. Many researchers have conducted studies on the polymerization processes of MIPs for the detection of different explosives, mainly focusing on nitroaromatic explosives, nitrate esters, nitroamine explosives, and peroxide explosives.

#### 2.2.1. Nitroaromatics

Nitroaromatic explosives are a class of aromatic compounds containing nitro (-NO_2_) substituents. The aromatic rings of these compounds have a stable conjugated π-electron system. Many high-energy nitroaromatic explosives (such as TNT and DNT) contain multiple nitro substituents, which are usually located at the ortho, meta, or para positions of the aromatic ring, forming symmetric or asymmetric structures. Certain nitroaromatic explosives may have additional substituents incorporated, such as a methyl group (-CH_3_) or an amino group (-NH_2_). These groups can influence the polarity and intermolecular forces of explosives. In MIP research related to explosives, studies on nitroaromatic explosives have been reported the most (as shown in Table 1). Among them, most studies focused on TNT.

Julia et al. [58] conducted theoretical calculations to explore the molecular interactions between 2,6-DNT and MAA complexes, providing a theoretical basis for selecting explosive molecule templates. In contrast to other researchers, their work was centered around theoretical research. Theoretical methods such as MP2/6-31G(d,p) and DFT/B3LYP were used to study the molecular structure, vibrational spectra, and interactions. It was clarified that MP2/6-31G(d,p) can be used to describe weak hydrogen-bond interactions, and DFT/B3LYP can be used to predict the vibrational properties of MIPs and template models. This study provided valuable methodological references for the theoretical calculations associated with the detection of explosives using molecular imprinting technology.

He et al. [59] synthesized D-A-type polytriphenylamine sulfone/ketone fluorescent polymers in their research and explored their applications in TNT detection, offering new ideas for the selection of functional monomers. They noted that functional monomers should have the ability to form specific interactions with explosive molecules, such as π–π stacking and hydrogen bonding interactions. These interactions can enhance the ability of a polymer to capture and recognize explosive molecules.

Gudiun Bunte et al. [42] investigated the trace detection of explosive vapors using MIPs. Their findings underscore the importance of choosing functional monomers capable of establishing stable interactions with template molecules. These interactions include hydrogen bonds, van der Waals forces, and electrostatic interactions. These interactions can strengthen the binding force between MIPs and explosive molecules, improving the recognition ability and selectivity of MIPs. Zhang Meijuan et al. [60] emphasized the importance of cross-linker selection in their research on detecting TNT via fluorescence quenching sensing technology. They noted that a suitable cross-linker should ensure that MIPs maintain a stable structure in a complex environment, thus maintaining their efficient ability to recognize explosive molecules.

Zhu Haoran et al. [61] further demonstrated the potential of cross-linkers in enhancing the performance of MIPs. They prepared a novel isoxazoline-based TNT adsorbent. By selecting an appropriate cross-linker, they not only improved the stability of the adsorbent but also enhanced its capacity to selectively adsorb TNT.

Puttasakul et al. [45] uniformly dispersed multiwalled carbon nanotubes (MWCNTs) in MIPs through sonication to form MIP-MWCNT composites. MWCNTs have excellent electrical conductivity and a large specific surface area. Adding them to MIPs can significantly improve the electrochemical performance of sensors. Experiments demonstrated that the high electrical conductivity of MWCNTs can accelerate electron transfer, thereby increasing the intensity of detection signals. Additionally, the large specific surface area of MWCNTs increases the number of active sites. This, in turn, contributes to enhancing the TNT adsorption capacity of sensors and improving their detection sensitivity.

Hassanzadeh et al. [46] combined zinc oxide QDs (ZnO QDs) with MIPs. ZnO QDs have good optical properties, a large specific surface area, and low toxicity. As carriers, they provide more attachment sites for MIPs and enhance the detection signal. MIPs endow the composite material with a specific recognition ability for TNT. The integration of the two types of materials capitalizes on the benefits of ZnO QDs in chemiluminescence detection and the high selectivity of MIPs, improving the detection performance. When preparing MIPs, the researchers used APTES as the functional monomer and TEOS as the cross-linker. The amino group in APTES can form multiple interactions with TNT, such as hydrogen bonds and electrostatic interactions, guiding the arrangement of functional monomers around TNT during the polymerization process. TEOS undergoes hydrolysis and polycondensation reactions, leading to the formation of a stable three-dimensional network structure. This structure effectively fixes the spatial configuration formed by the template molecule and functional monomers, enhancing the stability and recognition capability of MIPs.

In the three reports on the detection of 4-nitrophenol (4-NP), researchers used different functional monomers. The selection of functional monomers is closely associated with the characteristics of the template molecules, the nature of the detection targets, and the signal transduction methods employed. The functional monomer significantly influences the performance of MIPs, particularly in terms of their selectivity and sensitivity. For example, Chegel et al. [55] selected acrylamide (AAM) as the functional monomer because the amide group in the molecular structure of AAM can form hydrogen bonds or other weak interactions with the nitro and phenolic hydroxyl groups of 4-NP, forming a specific spatial structure and recognition sites around the template molecule during polymerization. This interaction gives MIPs a good specific recognition ability for 4-NP. Dai et al. [56] used a coumarin-based alkenyl fluorescent ionic liquid (coumarin-FL-IL) as the functional monomer. The coumarin structure exhibits intrinsic fluorescence characteristics. It can participate in electron transfer with 4-NP, causing fluorescence quenching, which is utilized in fluorescence detection. Moreover, the imidazole group in the ionic liquid can generate electrostatic and π–π interactions with the aromatic ring of 4-NP, enhancing the adsorption and recognition of the template molecule. Amiripour et al. [57] chose MAA as the functional monomer. The carboxyl group in MAA can establish hydrogen bonds with the hydroxyl and nitro groups of 4-NP. Additionally, the double bond in MAA can participate in the polymerization reaction. As a result, a stable MIP layer is formed on the surface of the amine-functionalized metal–organic framework (amine-UiO-66). The selection of MAA not only ensures the specific recognition of 4-NP but also does not affect the energy transfer efficiency in the Förster resonance energy transfer (FRET) process, ensuring the accuracy of detection.

Dai et al. [53] developed an amino-functionalized carbon dot (AC-dot)-labeled MIP sensor, overcoming the environmental toxicity problem caused by the potential dissolution of heavy metals in QDs (Q-dots). In addition, compared with the coated carbon QD (C-dot) system, the AC-dot film is easier to operate and reduces the interference of nonspecifically adsorbed compounds.

Bird et al. [49] used molecular dynamics to simulate the complexation process of TNT and MAA in different porogens. An analysis of the radial distribution function (RDF) of MAA-MAA, Kirkwood–Buff integral (KBI), and cluster size revealed that a binary porogen with an acetonitrile-to-dimethyl sulfoxide molar ratio of 75:25 had the greatest effect on disrupting the aggregation of MAA, which could increase the probability of complexation between TNT and MAA. Water significantly reduces the degree of aggregation of MAA, which might decrease the sensitivity for TNT detection in water based on MIPs. Therefore, a binary porogen with an acetonitrile-to-dimethyl sulfoxide molar ratio of 75:25 is suitable for synthesizing more efficient TNT-MIPs. When detecting TNT in an aqueous medium, a more suitable functional monomer than MAA should be selected.

Lu et al. [62] prepared molecularly imprinted colloidal particles (MICs) with excellent TNT adsorption performance. The adsorption capacity reached 64 mg/g, and the imprinting efficiency was high, with an equilibrium binding constant Ka of 2.88 L/mmol. Compared with nonimprinted colloidal particles (NICs), the MICs have a stronger affinity for TNT.

#### 2.2.2. Nitrate Esters

Nitrate ester explosives are a class of compounds containing a nitrate ester group (-ONO_2_). The nitrate ester group has strong polarity, making these explosive molecules prone to forming hydrogen bonds and other interactions with polar solvents or functional monomers. In MIP research related to explosives, there are relatively few reports on nitrate ester explosives (as shown in Table 2).

Yang et al. [63] studied the preparation of MIPs for detecting nitrocellulose (NC) and their applications in detection. In this study, the preparation temperature and ratio of functional monomers to cross-linkers were changed, and the functional group structure, surface morphology, crystal structure, and thermal stability of the MIPs, as well as the specificity and selectivity of the MIPs for NC, were investigated through Fourier transform infrared (FT-IR) spectroscopy, scanning electron microscopy (SEM), X-ray diffraction (XRD), thermogravimetric analysis (TGA), and UV–vis spectroscopy. Meng et al. [64] prepared SiO_2_/MIPs for the detection of NC and optimized the polymerization reaction conditions. The experiments revealed that the optimal reaction temperature was 45 °C, and the best ratio of MAA to EGDMA was 1:3.

#### 2.2.3. Nitramines

Nitroamine explosives are a class of compounds containing a nitroamine group (-N-NO_2_). Many nitroamine explosives (such as 1,3,5-trinitroperhydro-1,3,5-triazine (RDX) and octahydro-1,3,5,7-tetranitro-1,3,5,7-tetrazocine (HMX)) have a nitrogen-containing heterocyclic structure, in which the nitrogen atoms in the ring are connected to nitro groups, forming stable high-energy compounds. The nitroamine group also has strong polarity, making these explosive molecules prone to forming hydrogen bonds and other interactions with polar solvents or functional monomers. In MIP research related to explosives, there are relatively few reports on nitroamine explosives (as shown in Table 3).

Wang Jian et al. [40] used HMX and RDX as templates and prepared MIPs via precipitation polymerization. They prepared various MIPs with different ratios of templates, monomers, and cross-linkers, including 1:4:20 and 1:8:8. HMX and RDX are insoluble in most common organic solvents. Considering solubility, these researchers selected acetonitrile as the solvent, which is conducive to the formation of polar interactions between functional monomers and templates during the molecular imprinting process. A low temperature is generally believed to increase the stability of the complexes formed in the molecularly imprinted prepolymerization mixture. Wang Jian et al. reported that MIPs prepared by UV-induced polymerization at a lower temperature (4 °C) had a better imprinting effect than those prepared by thermal initiation (60 °C). Therefore, various MIPs for HMX and RDX detection were prepared under UV irradiation at 4 °C.

In another study by Wang Jian et al. [40], CL-20 was used as the template, and multiple MIPs were prepared under different functional monomer and polymerization conditions. The imprinting effects of the MIPs prepared under different imprinting conditions were compared, and the mechanism was analyzed. The MIPs with the best imprinting effect were screened out. Experiments revealed that when AAM was used as the functional monomer, ethylene glycol dimethacrylate (EGDMA) was used as the cross-linker, and the ratio of the template, functional monomer, and cross-linker was 1:6:24, the prepared MIPs had the highest adsorption capacity and adsorption selectivity for CL-20 in acetonitrile. The maximum adsorption capacity of MIPs for CL-20 was 115.7 mg/g, and adsorption equilibrium could be reached in 10 min. The researchers subsequently used these MIPs for the selective SPE of CL-20 from soil samples containing tetraacetyldibenzylhexaazaisowurtzitane (TADB), TNT, RDX, HMX, and CL-20, enabling HPLC-based quantitative detection.

Wang et al. [66] employed CL-20 as a template to fabricate MIHSs via a series of steps, including silica surface modification, surface imprinting, silica removal, and template removal. In parallel, nonimprinted polymer hollow spheres (NIHSs) were prepared as a control. They studied the binding properties of MIHSs and NIHSs via the batch method, optimized the SPE conditions, and evaluated the purification efficiency of MIHSs with simulated post-explosion samples.

#### 2.2.4. Peroxides

Peroxide explosives are a class of compounds containing a peroxide group (-O-O-). Common peroxide explosives, such as triacetone triperoxide (TATP) and hexamethylene triperoxide diamine (HMTD), typically feature an organic molecule, such as acetone or hexamethylenetetramine, as their structural backbone. In these explosives, hydrogen atoms are replaced by peroxide groups. The peroxide groups of these compounds are highly reactive and unstable. Compared with other types of explosives, the application of MIPs in the analysis of peroxide explosives is relatively limited (as shown in Table 4).

Mamo et al. [67] and Saglam et al. [68] both focused on the electrochemical detection of TATP and used MIP technology to improve the detection performance. Mamo et al. utilized pyrrole as the functional monomer. In a lithium perchlorate (LiClO_4_) electrolyte, pyrrole and TATP template molecules were electrochemically polymerized via cyclic voltammetry. This process led to polymerization of pyrrole on the electrode surface, thereby forming a polymer with TATP-imprinted sites. Moreover, they optimized the monomer concentration, template concentration, and polymerization conditions to achieve the electrochemical detection of TATP. Saglam et al. selected carbazole as the monomer. Through cyclic voltammetry, they electrochemically polymerized carbazole in a solution that contained the template molecule (either TATP or HMTD) and tetraethylammonium perchlorate (TEAP) as the supporting electrolyte. This reaction resulted in the formation of a peroxide-based explosive (PBE)-memory-polycarbazole (PCz) film.

In summary, in the realm of explosive analysis, MIPs can be synthesized via a plethora of methodologies, each exhibiting distinctive merits and demerits. Among the traditional preparation techniques, the entrapment method is widely used, but it presents issues such as complex post-treatment, easy destruction of imprinted cavities, material waste, the low utilization rate of binding sites, and the need to add a large amount of chemical reagents. New technologies have effectively overcome some of the drawbacks of traditional techniques and demonstrated unique advantages. These technologies have significantly enhanced the performance of MIPs and broadened their application scope. Concurrently, notable discrepancies exist in the selection of crucial experimental parameters, including template molecules, functional monomers, cross-linkers, and solvents, when fabricating MIPs tailored for diverse categories of explosives. Taking the porogen as an example, the types of porogens for nitroaromatic explosives are diverse. For nitrate ester explosives, acetone is predominantly employed. In the case of nitramine explosives and peroxide explosives, acetonitrile serves as the primary porogen. These parameters have a significant impact on the morphology, chemical properties, and recognition performance of MIPs. The preparation methods of MIPs in explosive analysis are constantly evolving, laying a solid foundation for subsequent applications in explosive detection, separation, and enrichment.

## 3. Applications of Molecularly Imprinted Polymers in the Analysis of Explosives

### 3.1. Sample Pretreatment

#### 3.1.1. Solid-Phase Extraction

SPE is one of the most common pretreatment techniques for MIPs used in the analysis of explosives [69]. In the detection of explosive residues, through the preparation of MIPs specific to certain explosive molecules and their utilization as selective SPE adsorbents, target explosive molecules can be selectively extracted from complex samples such as soil, water, and air particulate matter. This approach enables the selective adsorption and enrichment of explosive residues within samples. MIPs have been widely applied in this field [70,71,72].

Most research subjects are nitroaromatic explosives. Meng et al. [73] designed, prepared, and tested MIPs for the removal of TNT from “pink water”. They used suspension polymerization to prepare MIP microspheres with an average diameter of 25 µm, and the imprinting effect was 4.3. In the removal of TNT from “pink water”, the MIPs were used as the adsorption material for SPE. After SPE, TNT was concentrated from many “pink water” samples into a small amount of eluent, resulting in a relatively pure and high-concentration TNT solution. The experimental results revealed that the absorption rate of the MIP adsorbent was 65 times higher than that of granular activated carbon. In addition, the MIP adsorbent was easily regenerated, and the capacity decreased by only 7% after four rounds of regeneration.

Lordel et al. [74] synthesized molecularly imprinted silica adsorbents via the sol–gel method and used the prepared adsorbents for the extraction of nitroaromatic explosives within different sample matrices. They investigated the selectivity, adsorption capacity, adsorption kinetics, and thermodynamics of adsorbents for different nitroaromatic explosives. Moreover, comparisons were made with nonimprinted silica adsorbents and other traditional adsorbents. The experimental results revealed that under certain conditions, the molecularly imprinted SPE (MISPE) method had a high adsorption capacity for nitroaromatic explosives and could effectively enrich low-concentration explosives. The adsorption kinetics study also revealed that the adsorption process reached equilibrium in a relatively short time.

With respect to other types of explosives, although many studies have been conducted on the preparation of MIPs, few studies have focused on the corresponding MISPE procedures and the treatment of actual samples. Wang et al. [75] synthesized MIPs using CL-20 as the template molecule, filled the synthesized MIPs into an SPE column, and prepared an MISPE column for SPE of CL-20. After the soil sample was pretreated, the sample solution was passed through the MISPE column. CL-20 bound to the specific recognition sites on the MIPs, whereas other impurities flowed out with the mobile phase. The column was subsequently eluted with an eluent to elute CL-20 from the MIPs. Through comparative experiments, these researchers compared the MISPE method with traditional SPE methods and other common CL-20 extraction methods. The experimental results indicated that the synthesized MIPs exhibited a high level of selectivity for CL-20. These compounds could effectively differentiate CL-20 in soil from other structurally analogous compounds and impurities. As a result, the MIPs successfully accomplished specific extraction of CL-20 from the complex soil matrix. Moreover, the MISPE column had a high adsorption capacity for CL-20, effectively enriching low-concentration CL-20 within soil and meeting the requirements of trace analysis. Wang et al. [75] also conducted spiked recovery experiments on actual soil samples to evaluate the accuracy and reliability of the method. In the spiked recovery experiments conducted on actual soil samples, the recovery rate of CL-20 was notably high, whereas the relative standard deviation (RSD) was small. This outcome demonstrated that the method possessed excellent accuracy and reproducibility, enabling the precise determination of CL-20 content in soil.

RDX is the main byproduct of the synthesis of HMX. Owing to their similar sizes and physical properties, separating them is difficult. However, in another study by Wang et al. [76], they synthesized MIPs using HMX or RDX as the template molecule and filled the synthesized MIPs into an SPE column to prepare an MISPE column. They passed a mixed sample solution containing HMX and RDX through the MISPE column and utilized the specific recognition ability of MIPs for the template molecules to achieve the effective separation of HMX and RDX. Moreover, under optimized conditions, the MISPE column effectively enriched HMX and RDX within low-concentration samples. The adsorption capacity for the target substances was significantly greater than that of NIP columns and other control columns.

The MISPE technique is an efficient and highly selective separation method that can be used for the separation and enrichment of explosives. It has potential application value in fields such as environmental monitoring and analysis of explosives. Compared with traditional solvent separation methods, MISPE technology has greater selectivity, sensitivity, and recovery rates and has the advantages of complete separation, environmental friendliness, and being operable at room temperature. Moreover, MISPE technology is convenient for the quantitative analysis of explosives using various analytical methods, such as chromatography and spectroscopy, and it provides more favorable conditions for the further treatment or degradation of explosives.

#### 3.1.2. Solid-Phase Microextraction

Solid-phase microextraction (SPME) is a sample pretreatment technique that integrates sampling, extraction, concentration, and injection, and it has broad application prospects [77]. The combination of MIPs and SPME technology, in which MIPs are immobilized on an extraction fiber either by chemical bonding or physical coating, not only streamlines the operation procedures but also increases the sensitivity and precision of the analysis [78,79]. In the field of analysis of explosives, Bianchi et al. [80] synthesized an MIP using TNT as the template molecule and used it as a new coating for the selective determination of 2,4,6-trinitrotoluene in nitroaromatic explosives via SPME. The fibers were characterized in terms of the coating thickness, morphology, intrabatch and interbatch repeatability, and extraction efficiency. The average thickness of the molecularly imprinted fiber coating was 50 ± 4 μm, with a uniform distribution. This method had good performance in terms of intrabatch and interbatch repeatability, with an RSD < 8%. In the experiment, detection and quantification limits at the low nanogram/kilogram level were achieved, demonstrating the excellent extraction ability of the developed coating. Moreover, the obtained gas chromatography–mass spectrometry analysis response was approximately twice that obtained when using commercial equipment.

There are relatively few specific application cases of using MIP-SPME in the analysis of explosives, but its successful application in other fields, such as environmental analysis and food analysis, can provide useful references for the analysis of explosives.

#### 3.1.3. Liquid-Phase Microextraction

Liquid-phase microextraction (LPME) is a new type of sample pretreatment technique developed on the basis of liquid-phase extraction. In the field of analysis of explosives, Ebrahimzadeh et al. [81] established a method for determining nitrotoluenes in wastewater by using a gas chromatography-flame ionization detector combined with MIPs and dispersive liquid–liquid microextraction (DLLME). MIPs were copolymerized and synthesized using azobisisobutyronitrile (AIBN) as the initiator and 3-nitrotoluene as the template molecule for imprinting. These researchers first optimized the extraction process of 3-nitrotoluene in MIPs and then optimized DLLME. Under the optimal conditions, the enrichment factor of the MIP-DLLME method was approximately 2800. The detection limit of this method was 0.02 μg/L, and the linear dynamic range was 0.04–20 μg/L. The performance of this method in the extraction and determination of nitrotoluene compounds in wastewater samples within the microgram/liter range was evaluated, and satisfactory results were obtained (RSD < 13%).

Although the use of MIP-LPME has not been widely reported in the field of explosives’ analysis, its successful application in other fields [82,83] indicates that LPME combined with MIP technology has the potential to become a new and efficient pretreatment method for the analysis of explosives. LPME has the advantages of low solvent consumption, simple operation, and high enrichment efficiency [84]. By leveraging the high selectivity and specificity inherent in MIPs and optimizing extraction conditions, MIP-LPME technology is anticipated to play a crucial role in the analysis of explosives. This technology holds promise for enabling efficient extraction and detection of trace explosive molecules.

### 3.2. Sensors

As sensitive elements of sensors, MIPs are generally located on the surface of the transducer element and at the interface with the analyte. When the target analyte undergoes a bonding reaction with MIPs, the transducer element accurately expresses and successfully transmits the signal. MIPs, as recognition elements, have the following advantages: (1) they are selective and can specifically bind to the target substance within complex analytes; (2) they are resistant to acids and alkalis, not easily corroded, inexpensive, easy to prepare, and convenient to store; (3) the detection signals can be replicated and amplified; and (4) they have a certain degree of stability and can be repeatedly used. Currently, molecularly imprinted sensors are being extensively researched for use in areas such as pharmaceutical analysis, pollutant detection, and food additive detection. Moreover, they exhibit significant application potential in the field of explosives’ detection [85]. In accordance with their different response signals, MIP sensors mainly include electrochemical sensors and photochemical sensors.

#### 3.2.1. Electrochemical Sensors

Among the various sensors, MIPs were first used in the preparation of electrochemical sensors. As early as 1993, Hedborg et al. [86] combined an MIP membrane with a field-effect device as a sensor to monitor electrochemical signals. MIPs can be combined with electrochemical sensors to prepare highly selective and sensitive explosive detection sensors [87] (as shown in Table 5).

Samuel et al. [67] developed an MIP-based sensor for the electrochemical determination of TATP. They successfully prepared MIPs with a high degree of selectivity for TATP by optimizing the preparation process and demonstrated their potential application in the detection of explosives. Shi et al. [94] reported an effective strategy for recognizing TNT molecules on the surface of a graphene–polyaniline (PANI) nanocomposite. Using picric acid as the template analog of the explosive for imprinting template synthesis, TNT was detected via differential pulse voltammetry and cyclic voltammetry.

In 2020, Leibl et al. [88] developed a sensitive electrochemical sensor based on a molecularly imprinted polydopamine film for the detection of nitro explosives in aqueous solutions. Based on density functional theory (DFT) calculations, dopamine was determined to be the best functional monomer on silicon, and electropolymerization was performed on two nitro explosives, TNT and RDX, via cyclic voltammetry. This method provided a uniformly covered gold electrode with an adjustable-thickness imprinted film. The electropolymerized molecularly imprinted polydopamine film enabled the redox peaks of the target to be tracked via cyclic voltammetry, increasing the sensitivity by 105 times compared with that for the bare gold electrode. The MIP film could achieve repeatable binding in phosphate-buffered saline (10 mM, pH 7.4). The dynamic range for TNT and RDX was 0.1 nmol/L to 10 nmol/L, with high selectivity.

Sağlam et al. [89] developed a glassy carbon sensor electrode modified with a gold nanoparticle/poly(carbazole–aniline) film, enabling the simultaneous detection of multiple nitroaromatic and nitroamine explosives with the help of MIPs. At the same time, they comprehensively studied the performance of this sensor electrode, covering aspects such as the detection ability, selectivity, and sensitivity, and they evaluated its feasibility for use in actual sample detection. The results showed that this sensor exhibited good application potential in the detection of explosives. Shahdost-Fard et al. [90] also used a glassy carbon sensor to detect TNT. The difference was that Shahdost-Fard et al. used a glassy carbon electrode modified with a gold nanoparticle@fullerene (AuNP@C60) composite. Fullerene improved the material stability and biocompatibility, promoted the fixation of aptamers and polymers, and enhanced the TNT recognition ability.

The sensor prepared by Mamo et al. [67] had a detection limit of 26.9 μg/L and a detection range of 82–44,300 μg/L. In the study by Saglam et al. [68], the detection limit for TATP was 15 μg/L, and the detection range was 100–1000 μg/L. In comparison, the sensor developed by Saglam et al. had a lower detection limit and a different detection range, reflecting an improvement in the detection sensitivity. Moreover, from the perspective of the integrity of the detection object, the study by Mamo et al. did not involve actual sample analysis or the determination of PBEs in the presence of H_2_O_2_. A study by Saglam et al. revealed that intact TATP and HMTD could be directly detected without interference from H_2_O_2_ and could be detected in multiple complex environments.

Tancharoen et al. [54] overcame the limitations of traditional single-molecule imprinting and introduced the coimprinting of dengue virus and DNT. Owing to the binding properties between the virus surface and DNT, special recognition sites were formed in the polymer, significantly enhancing the selectivity and sensitivity of the sensor for explosive molecules. Multiple explosives could be effectively distinguished, and the sensor showed a good linear relationship within four orders of magnitude, outperforming traditional MIP sensors.

Guo et al. [31] combined MIPs, a metal–organic framework (MMOF), and gold nanoparticles (AuNPs). The MIPs provided the specific recognition ability for TNT. The MMOF, owing to its unique electronic properties and interactions with nitroaromatics, enhanced detection sensitivity and selectivity. The AuNPs improved the conductivity of the electrode surface, increased the electron transfer rate, and promoted the uniform distribution of recognition sites in the MIP film. The synergy of these three materials enhanced the sensor performance. This design idea of multimaterial composites provides a new direction for the development of new chemical sensors.

Riskin et al. [92] modified the electrode surface to introduce π-donor–acceptor interactions, thus enriching TNT on the electrode surface. The detection limit of the p-aminothiophenol-modified gold electrode reached 17 μg/L (74 nmol/L), the detection limit of the oligoaniline-cross-linked Au NP-modified electrode was as low as 460 ng/L (2 nmol/L), and the detection limit of the picric acid-imprinted oligoaniline-cross-linked Au NP-modified electrode was further reduced to 46 ng/L (200 pmol/L). Compared with traditional detection methods and unmodified electrodes, the TNT detection sensitivity was greatly improved.

Trammell et al. [93] established a detection system with significantly improved TNT detection performance. When the sample volume was 2 mL, the detection limit was as low as 13 μg/L, and the linear range was 20–500 μg/L. When 480 mL of the sample (buffer solution or seawater) was preconcentrated, 0.5 μg/L TNT could be detected, with a signal-to-noise ratio of 20. The detection limit was significantly lower than that of previous methods, and TNT within complex matrices (such as seawater) could be effectively detected, expanding the application range of detection.

#### 3.2.2. Photochemical Sensors

The application of MIPs in photochemical sensors occurred later than that in electrochemical sensors, but it has been widely studied thus far. For example, photochemical sensors based on MIPs have been applied in the detection of anti-inflammatory drugs, organophosphorus pesticides, and other substances [95,96,97]. In the analysis of explosives, photochemical sensors achieve detection by utilizing changes in optical signals after MIPs bind to target explosives. Common types of photochemical sensors include fluorescence sensors and surface-enhanced Raman spectroscopy (SERS) sensors, among others (as shown in Table 6).

Among them, fluorescence-type sensors have attracted increasing attention because of their low detection limits. When MIPs with fluorescence signals bind to the detected substances, their fluorescence signals change. In the field of analysis of explosives, fluorescent groups are introduced into MIPs or fluorescently labeled target explosive molecules are used in fluorescence sensors. When the two materials combine, the fluorescence intensity or wavelength changes, enabling the detection of explosives.

Huynh et al. [104] newly prepared a 3D MIP membrane. This membrane contains a unit for recognizing TNP, and a fluorophore for signal transduction and quantification is embedded in it. In this way, it shows selectivity for and sensitivity to TNP analytes in solution. The experimental results showed that the detection limit of this chemical sensor reached the sub-nanogram-per-liter level. In addition, this MIP membrane was prepared via the one-step electropolymerization of a prepolymerization solution. Therefore, this method is easy to scale up and can be used for the selective determination of other nitroaromatic explosives.

Dai et al. [56] utilized the fluorescence quenching principle of fluorescent molecularly imprinted polyionic liquids to detect 4-NP through changes in fluorescence signals. The fluorescence sensor of Amiripour et al. [57] was a core–shell-structured nanocomposite formed by an amine-functionalized metal–organic framework (MOF) and an MIP. Based on the FRET mechanism, 4-NP was determined by detecting changes in the fluorescence intensity. By comparing the two studies, the advantages of Dai et al.’s method were found to be that the material itself is a fluorescent polymer, the preparation process is relatively simple, the material can respond quickly in liquid samples, and owing to the hydrophilicity of polyionic liquids, it has good compatibility with the aqueous phase and can efficiently detect 4-NP in a water environment. The advantages of the method of Amiripour et al. are that it combines the high adsorption capacity of MOFs and the high selectivity of MIPs and can effectively remove interfering substances in complex matrices to achieve trace detection.

Cennamo et al. [99] utilized five-branched gold nanostars (GNSs) to replace traditional spherical gold nanoparticles. The GNSs were then dispersed in TNT-specific MIPs to create a GNS-MIP sensing layer, which was directly deposited on two platforms as follows: a plastic optical fiber (POF) and a tapered POF. The experimental results showed that the sensitivity of the GNS-MIP-based sensor for detecting TNT was significantly improved. The sensitivity of the GNS-MIP sensor on the nontapered POF was 3 times greater than that of the traditional gold-layer sensor, and the sensitivity of the GNS-MIP sensor on the tapered POF reached 30 times that of the traditional gold-layer sensor. The detection limit was also greatly reduced while maintaining the high selectivity of MIPs for TNT.

Della Giustina et al. [100] combined MIPs with sol–gel materials. The low-temperature processing conditions and versatility of sol–gel materials make them an ideal matrix for MIPs. The molecularly imprinted sol–gel matrix formed by their combination had high selectivity and could specifically recognize TNT molecules. Moreover, the researchers used azimuth-controlled grating-coupled SPR technology to greatly improve the detection sensitivity. This sensing platform could detect trace TNT gases as low as below 1 μg/L, with a sensitivity of 0.47°/μg/L. Moreover, this platform has a low cost and can be miniaturized, showing great potential in practical applications such as airport security.

Stringer et al. [22] compared chloroform-based and water-based systems and reported that MIP microspheres prepared with chloroform as the solvent had better performance in detecting nitroaromatics. The chloroform-based microspheres were spherical and uniform in size, with a specific surface area of 21 m^2^g^−1^. The detection limits for TNT and DNT were as low as 0.1 μmol/L and 0.01 mmol/L, respectively, and the response times were short. The fluorescence intensity significantly decreased within 1 min when detecting TNT. In contrast, the water-based particles were composed of interconnected nanospherical structures. Although the specific surface area was 34.52 m^2^g^−1^, the detection limits for TNT and DNT were relatively high, at 0.1 mmol/L and 0.02 mmol/L, respectively. This discovery reveals the crucial influence of the polymerization solvent on the performance of MIP materials and provides an experimental basis for screening suitable polymerization solvents.

Xu et al. [24] focused on the application of dummy template molecular imprinting technology to solve problems such as template leakage. They prepared a fluorescence sensor of dummy MIP-coated CdTe QDs (DMIP@QDs), and the detection signal was generated based on the principle of electron-transfer-induced fluorescence quenching. In another study by Xu et al. [102], they focused on the research and application of the dual-signal amplification mechanism to optimize the detection performance. They constructed a fluorescence sensor based on MIP@QDs, adopting a dual-signal amplification strategy of ratio fluorescence and a mesoporous structure. The use of ratio fluorescence can reduce background interference, and the mesoporous structure can increase the amount of TNT adsorbed. In 2016, Xu et al. [103] focused on the design and performance optimization of the mesoporous structure to improve the sensor sensitivity. They prepared a mesoporous-structured MIP@carbon dot (CD) fluorescence sensor. Its large specific surface area and appropriate pore size were conducive to the diffusion and adsorption of TNT, resulting in signal amplification.

SERS sensors, with the help of the specific recognition of MIPs and the high sensitivity of SERS, can quickly and accurately detect trace explosives. Holthoff et al. [98] employed a sol–gel-derived xerogel as the base material to fabricate MIPs. They harnessed SERS technology in an integrated transduction approach to increase sensor performance. Thus, they overcame the issue of nonspecific binding in MIPs and developed a sensitive and selective MIP-SERS sensing platform for detecting TNT. In 2024, the research group of Professor Han Sheng [106] reported emerging construction strategies and sensing mechanism research methods for sensors combining surface MIPs and SERS (SMIPs-SERS) and introduced their practical applications and development trends to hazardous substance analysis.

Lu et al. [62] developed a visual detection method based on molecularly imprinted colloidal array (MICA) photonic crystals to directly detect TNT through the diffraction color change in the MICA. This sensor exhibited excellent selectivity, showing a significantly greater response to TNT than to a variety of similar compounds. This sensor demonstrated remarkable stability, with a diffraction change of less than 2 nm when detecting 5 mmol/L TNT, even after three years of storage. Additionally, it had good reversibility, with a standard deviation of only 3% after multiple detection cycles.

#### 3.2.3. Other Sensors

In addition to electrochemical sensors and photochemical sensors, MIPs can also be combined with other types of sensors.

Fan et al. [107] developed a molecularly imprinted photonic crystal (MIPC) sensor for detecting typical nitroamine explosives such as RDX, HMX, CL-20, and TNT. The experiments revealed that as the concentration of explosives increased, the diffraction peak and structural color of the MIPC sensor redshifted within 5 min. After an alkaline solution was added, the MIPC sensor softened, and its mass transfer and sensitivity improved. This sensor can respond to explosives at concentrations of less than 0.05 mmol/L, and the detection limit is 0.01 mmol/L. The results showed that semiquantitative detection could be achieved by using the MIPC to construct an MIPC sensor array. Moreover, different explosive molecules could be distinguished by using the pattern recognition technique with a 48 × 4 multivariate data matrix.

Ni Yuan et al. [43] introduced a piezoelectric sensor based on MIPs for detecting explosives. They used MIPs as the recognition element of the sensor and a piezoelectric crystal as the transduction element to fabricate a molecularly imprinted piezoelectric sensor. The experimental results showed that the detection limit of this sensor for TNT could reach 10⁻⁶ g. This sensor has good sensitivity and repeatability and can be used for onsite detection, showing potential for use in security inspection departments such as those at airports and railway stations.

Apodaca et al. [108] prepared a simple and robust nitroaromatic compound sensor using the two-dimensional molecularly imprinted monolayer (2D MIM) method. They formed a 2D MIM on the gold-surface electrode of a quartz crystal microbalance (QCM) and used it to detect analytes at different concentrations. The experimental results showed that the QCM could selectively detect DNT, even in a mixed solution of competitive molecules, indicating the high-selectivity advantage of this method.

These sensors each have their own characteristics, and appropriate sensor types can be selected according to different detection requirements and sample characteristics. For example, mass-sensitive sensors can directly measure the mass change in explosive molecules, whereas photonic crystal sensors can achieve semiquantitative detection of explosives through the construction of arrays and their combination with pattern recognition.

### 3.3. Other Emerging Applications

In addition to the sample pretreatment and sensors mentioned above, MIPs have also shown potential for other emerging applications in the field of analysis of explosives. Researchers have further explored the application potential of MIPs in the analysis of explosives by combining them with technologies such as nanomaterials and mass spectrometry.

In recent years, nanomaterials have undergone rapid development and have received extensive attention in global scientific research and industrial fields. Nanomaterials possess advantages such as high activity, high strength, and high toughness. Owing to these advantages, nanomaterials have found extensive applications across diverse fields, including in the energy, medical, and environmental fields. For example, they are used to increase battery performance, enable the manufacturing of smaller chips, achieve targeted drug delivery, and purify sewage and air [109,110]. Nanomaterials are thus constantly propelling technological innovation in multiple sectors. To further improve the performance of MIP-based sensors in the analysis of explosives, researchers have attempted to combine nanomaterials with MIPs.

Aznar-Gadea et al. [111] reported a solid-state luminescent gas sensor fabricated by embedding CsPbBr_3_ nanocrystals (NCs) into MIPs using 3-nitrotoluene (3-NT) and nitromethane (NM) as template molecules. The fabrication of the nano-MIP sensor is simple and cost-effective. The molecular imprinting process occurs within the CsPbBr_3_ nanocomposite in polycaprolactone (PCL). The sensing ability of the MIP sensor was evaluated by monitoring its photoluminescence (PL) and compared with that of the NIP. The response time of the nanocomposite sensor to analytes was less than 5 s. Experiments showed that NCs enhance the PL response of the MIP sensor, its specificity for 3-NT, and its good selectivity for nitro-containing molecules. Apak et al. [12] reported a piezoelectric sensor based on MIPs and nanomaterials. MIPs were coupled with nanomaterials such as graphene oxide (GOx), carbon nanotubes (CNTs), or nanoparticles (NPs) for the simultaneous detection of multiple explosives. By optimizing the sensor design, the highly selective detection of explosives such as TNT, RDX, and pentaerythritol tetranitrate (PETN) was achieved. In this paper, Apak et al. discussed the specific characteristics, modifications, and detection mechanisms of the nanomaterials involved in detail. Chegel et al. [55] focused on the field of detection of explosives and developed an efficient detection technique. The research team prepared a local surface plasmon resonance (LSPR) nanochip coated with MIPs for explosive sensing. The core of their research included the preparation of the nanochip, performance testing, and evaluation of its practical application potential.

Some researchers have also combined MIPs with mass spectrometry techniques. Cegłowski et al. [112] developed a new method for the selective detection and quantification of explosives using MIPs combined with flowing atmospheric pressure afterglow mass spectrometry (FAPA-MS). The experimental results showed the following: (1) The detection limit of this method is as low as 0.01 μmol/L. Compared with those obtained with direct solution analysis, the detection limits for picric acid and PETN are improved by two orders of magnitude, and that for RDX is improved by one order of magnitude. (2) Spiked river water samples have high recovery rates, within 8.2% of the true values, confirming the accuracy of the method. (3) This method has a strong linear relationship (R^2^ > 0.99) and good precision, with an RSD not exceeding 8.9%, further verifying the effectiveness of the system. By coupling MIPs with ambient plasma mass spectrometry, this method can sensitively, accurately, and reliably detect explosives. The rapid detection and quantitative analysis of explosives in water samples was achieved in the experiment, demonstrating the practical application potential of this method in environmental monitoring.

In conclusion, the applications of MIPs in the field of explosives’ analysis are constantly expanding. From sample pretreatment and sensors to nanotechnology and mass spectrometry, MIPs provide powerful technical support for the rapid and accurate detection of explosives. In the sample pretreatment stage, MIPs can effectively separate and enrich the target explosive molecules from complex samples due to their high selectivity. This application significantly improves the detection sensitivity and also has the advantages of reusability and environmental friendliness. In the field of sensors, MIPs, as sensitive elements, are combined with electrochemical, photochemical, and other technologies to construct various types of sensors. These sensors exhibit high sensitivity and selectivity in the detection of various explosives, with low detection limits and wide linear ranges. Some sensors also have good repeatability and stability. Additionally, integrating MIPs with nanomaterials and mass spectrometry has further exploited their potential in explosive analysis, enabling more efficient and precise detection.

## 4. Summary and Outlook

MIPs have demonstrated great application potential in the analysis of explosives. Through the meticulous design and preparation of MIPs, the highly selective recognition and high-efficiency adsorption of target explosive molecules can be accomplished. This allows successful implementations in areas such as sample pretreatment and sensors, thereby enabling the highly sensitive and selective detection of trace explosives. However, currently, most applications of MIPs in the analysis of explosives are aimed at single-target molecules. Achieving simultaneous and efficient detection for multicomponent explosive systems with similar structures is difficult, and versatility needs to be further improved.

In future research, on the one hand, efforts can be focused on developing new preparation techniques. New preparation techniques and methods, such as click chemistry [113,114,115] and 3D printing [116,117,118], can be explored to increase the imprinting efficiency, improve the structure and performance of MIPs, and achieve the efficient detection of multiple explosives in complex samples. On the other hand, the combination with other technologies can be expanded. The combination of MIPs with various advanced analytical techniques, such as mass spectrometry and capillary electrophoresis, can be intensified. Thus, the advantages of each technique can be fully utilized to achieve more accurate, rapid, and sensitive analytical detection of explosives.

## Figures and Tables

**Table 1:** Preparation methods for MIPs for detecting nitroaromatic-type explosives.

Target	Functional Monomer	Cross-Linking Agent	Porogen	Initiator	Initiation Method	Temperature	Preparation Method	Ref
TNT	MAA	Ethylene Glycol Dimethacrylate (EGDMA)	Chloroform	--	Thermal Initiation	50 °C	In situ Polymerization	[34]
TNT	Acrylate	EGDMA	Acetonitrile	UV-Starter Irgacure 369	UV Initiation	Room Temperature	In situ Polymerization	[42]
TNT	MAA	EGDMA	Chloroform	--	Thermal Initiation	70 °C	Surface Molecular Imprinting Method	[43]
TNT	MAA	EGDMA	Acetonitrile	Potassium Persulfate	Thermal Initiation	50 °C	Emulsion Polymerization	[44]
TNT	MAA	EGDMA	Dimethyl Sulfoxide (DMSO)	AIBN	--	--	Precipitation Polymerization	[45]
TNT	APTES	TEOS	Acetonitrile	--	--	Room Temperature	Surface Molecular Imprinting Method	[46]
TNT	MAA	EGDMA	Acetonitrile	AIBN	Thermal Initiation	60 °C	Surface Molecular Imprinting Method	[47]
TNT	AA	EGDMA	Acetonitrile	AIBN	Thermal Initiation	60 °C	Surface Molecular Imprinting Method	[48]
TNT	MAA	--	Acetonitrile (ACN), DMSO, Water, and Binary Mixtures of ACN and DMSO with Different Molar Ratios	--	--	25 °C	--	[49]
TNT	3-Aminopropyltriethoxysilane (APTES)	Tetraethyl Orthosilicate (TEOS)	Acetonitrile	--	--	--	Emulsion Polymerization	[24]
TNT	MAA	EGDMA	Acetonitrile	AIBN	Thermal Initiation	60 °C	Precipitation Polymerization	[50]
TNT	MAA	EGDMA	ACN and DMSO	AIBN	Thermal Initiation	65 °C	In situ Polymerization	[51]
TNT/DNT	MAA	EGDMA/EGDA	Chloroform	AIBN or 4,4′-Azobis(4-cyanovaleric Acid) (ACVA)	Thermal Initiation	50 °C	Precipitation Polymerization	[22]
TNT/DNT	AAM	EGDMA	ACN	ABVN	UV Initiation	4 °C	Precipitation Polymerization	[21]
TNT/DNT	MAA	EGDMA	Chloroform	1-Hydroxycyclohexylphenyl Ketone (Photoinitiator)	UV Initiation	--	Solution Polymerization	[52]
DNT	AA and MA	EGDMA	Methanol	AIBN	UV Initiation	Room Temperature	In situ Polymerization	[53]
DNT	MAA/NVP/MMA/AAM	N,N′-(1,2-Dihydroxyethylene)bisacrylamide (DHEBA)	DMSO	AIBN	UV Initiation	65 °C	Template-Virus Coimprinting Method	[54]
4-NP	AAM	--	--	--	UV Initiation	4 °C	Bulk Polymerization	[55]
4-NP	Coumarin-Based Alkenyl Fluorescent Ionic Liquid (Coumarin-FL-IL)	EGDMA	Methanol and ACN	--	--	--	Precipitation Polymerization	[56]
4-NP	MAA	EGDMA	Methanol	AIBN	--	--	Surface Molecular Imprinting Method	[57]

**Table 2:** Preparation methods for MIPs for detecting nitrate ester-type explosives.

Target	Functional Monomer	Cross-Linking Agent	Porogen	Initiator	Initiation Method	Temperature	Preparation Method	Ref.
NC	MAA	EGDMA	Acetone	AIBN	Thermal Initiation	50 °C	In situ Polymerization	[63]
NC	MAA	EGDMA	Acetone	AIBN	Thermal Initiation	45 °C	Surface Molecular Imprinting Method	[64]
NC	MAA	EGDMA	Acetone	AIBN	Thermal Initiation	55 °C	Surface Molecular Imprinting Method	[65]

**Table 3:** Preparation methods for MIPs for detecting nitramine-type explosives.

Target	Functional Monomer	Cross-Linking Agent	Porogen	Initiator	Initiation Method	Temperature	Preparation Method	Ref.
HMX and RDX	MAA/AAM	AIBN	Acetonitrile	AIBN	UV Initiation	4 °C	Precipitation Polymerization	[40]
CL-20	AAM	EGDMA	Acetonitrile	AIBN	Thermal Initiation	60 °C	Precipitation Polymerization	[40]
CL-20	AAM	EGDMA	Acetonitrile	--	UV Initiation	4 °C	Sacrificial Template Method	[66]

**Table 4:** Preparation methods for MIPs for detecting peroxide-type explosives.

Target	Functional Monomer	Cross-Linking Agent	Porogen	Initiator	Initiation Method	Temperature	Preparation Method	Ref.
TATP	Pyrrole	--	Acetonitrile	--	--	--	Electropolymerization (LiClO4)	[67]
TATP/HMTD	Carbazole (Cz)	--	Acetonitrile	--	--	--	Electropolymerization (TEAP)	[68]

**Table 5:** Detection effects of electrochemical sensors.

Target	LOD	Linear Range	Electrochemical Analysis Technique	Ref.
TNT	4.4 × 10^−17^ mol/L	4.4 × 10^−15^–4.4 × 10^−8^ mol/L	Linear Sweep Voltammetry (LSV)	[31]
TNT	5 × 10^−11^ mol/L	5 × 10^−11^–1.6 × 10^−8^ mol/L	Cyclic Voltammetry (CV)	[88]
TNT/DNT	1.1 × 10^−7^ mol/L/ 1.65 × 10^−7^ mol/L	4.4 × 10^−7^–4.4 × 10^−6^ mol/L/ 5.49 × 10^−7^–5.49 × 10^−6^ mol/L	Square Wave Voltammetry (SWV)	[89]
TNT	3.5 × 10^−8^ mol/L	1 × 10^−17^–1.5 × 10^−6^ mol/L	CV	[90]
DNT	4.4 × 10^−12^ mol/L	--	CV	[54]
TNT	1.32 × 10^−12^ mol/L	--	CV	[54]
TNT	5 × 10^−10^ mol/L	1 × 10^−9^–1.3 × 10^−7^ mol/L	SWV	[47]
TNT	6.2 × 10^−4^ mol/L/ 7 × 10^−5^ mol/L	7 × 10^−4^–5.6 × 10^−3^ mol/L	Chronoamperometry (CA) Piezoelectric Microgravimetry (PM)	[91]
TNT	2 × 10^−10^ mol/L	--	LSV	[92]
TNT	5.73 × 10^−8^ mol/L 2.2 × 10^−9^ mol/L	8.81 × 10^−8^–2.20 × 10^−6^ mol/L 2.20 × 10^−9^–2.20 × 10^−6^ mol/L	SWV	[93]
PETN	4.43 × 10^−12^ mol/L	--	CV	[54]
NC	3.45 × 10^−10^ g/L	0–7 × 10^−5^ g/L	CV and Differential Pulse Voltammetry (DPV)	[65]
RDX	1 × 10^−10^ mol/L	1 × 10^−10^–1.1 × 10^−8^ mol/L	CV	[89]
RDX/HMX	4.5 × 10^−8^ mol/L/ 3.4 × 10^−8^ mol/L	2.25 × 10^−7^–4.5 × 10^−6^ mol/L/ 1.69 × 10^−7^–3.38 × 10^−6^ mol/L	SWV	[90]
TATP	1.2 × 10^−7^ mol/L	3.69 × 10^−7^–2.0 × 10^−4^ mol/L	CV	[67]
TATP	6.75 × 10^−8^ mol/L	4.5 × 10^−7^–4.5 × 10^−6^ mol/L	CV and DPV	[68]
HMTD	8.61 × 10^−8^ mol/L	5.74 × 10^−7^–5.74 × 10^−6^ mol/L	CV and DPV	[68]

**Table 6:** Detection effects of photochemical sensors.

Target	LOD	Linear Range	Analysis Time	Sensor	Ref.
TNT	3 × 10^−6^ mol/L	--	--	Surface-Enhanced Raman Spectroscopy (SERS)	[98]
TNT	3.0 × 10^−11^ mol/L	8.8 × 10^−11^–2.2 × 10^−7^ mol/L and 2.2 × 10^−7^–8.8 × 10^−7^ mol/L	--	Chemiluminescence (CL)	[46]
TNT	1 × 10^−8^ mol/L	1 × 10^−8^–1 × 10^−5^ mol/L	--	Surface Plasmon Resonance (SPR)	[48]
TNT	4.1 × 10^−7^ mol/L	--	--	Localized Surface Plasmon Resonance (LSPR)	[99]
TNT	1.15 × 10^−12^ mol/L	--	--	SPR	[100]
TNT	1.03 × 10^−6^ g	3 × 10^−4^–3 × 10^−2^ mol/L	120 s	Photonic Crystal (PhC)	[62]
TNT	4.40 × 10^−7^ mol/L	2.20 × 10^−6^–2.20 × 10^−4^ mol/L	--	IMS	[21]
TNT	Chloroform-based: 1 × 10^−7^ mol/L Water-based: 1 × 10^−4^ mol/L	--	60 s	Fluorescence	[22]
TNT	4.07 × 10^−5^ mol/L	0–5 × 10^−4^ mol/L	60 s	Fluorescence	[52]
TNT	3.74 × 10^−11^ mol/L	1.76 × 10^−11^–4.40 × 10^−11^ mol/L	100 s	Optical Waveguide Spectroscopy (OWS)	[101]
TNT	2.8 × 10^−7^ mol/L	8 × 10^−7^–3 × 10^−5^ mol/L	--	Fluorescence	[24]
TNT	1.5 × 10^−8^ mol/L	5 × 10^−8^–6 × 10^−7^ mol/L	--	Fluorescence	[102]
TNT	1.7 × 10^−8^ mol/L	5 × 10^−4^–2 × 10^−2^ mol/L	--	Fluorescence	[103]
TNT	1 × 10^−10^ mol/L	1 × 10^−15^–1 × 10^−7^ mol/L	--	SPR	[51]
DNT	3.01 × 10^−5^ mol/L	0–5 × 10^−4^ mol/L	600 s	Fluorescence	[52]
DNT	Chloroform-based: 1 × 10^−5^ mol/L Water-based: 2 × 10^−5^ mol/L	--	300 s	Fluorescence	[22]
DNT	2.75 × 10^−7^ mol/L	5.49 × 10^−7^–5.49 × 10^−5^ mol/L	--	IMS	[21]
TNP	8.73 × 10^−13^ mol/L	8.73 × 10^−13^–8.91 × 10^−11^ mol/L	--	Fluorescence	[104]
DNT	1.54 × 10^−6^ mol/L	5.49 × 10^−6^–8.24 × 10^−5^ mol/L	1800 s	Fluorescence	[53]
4-NP	5 × 10^−10^ mol/L	1 × 10^−9^–7.5 × 10^−6^ mol/L	60 s	Fluorescence	[56]
4-NP	9 × 10^−9^ mol/L	5 × 10^−8^–5 × 10^−5^ mol/L	--	Fluorescence	[57]
RDX	2 × 10^−10^ g	--	20 s	Fluorescence	[105]
HMX	3 × 10^−10^ g	--	20 s	Fluorescence	[105]
PETN	3 × 10^−10^–3 × 10^−9^ g	--	20 s	Fluorescence	[105]

## Abbreviations

The following abbreviations are used in this manuscript:

MIPs	Molecularly Imprinted Polymers
HPLC	High-performance Liquid Chromatography
CEC	Capillary Electrochromatography
SPE	Solid-Phase Extraction
NIPs	Nonimprinted Polymers
TNT	Trinitrotoluene
IMS	Ion Mobility Spectrometry
QDs	Quantum Dots
MMA	Methyl Methacrylate
NVP	Nvinylpyrrolidone
MAA	Methacrylic Acid
MA	Methyl Acrylate
AA	Acrylic Acid
TRIM	Trimethylolpropane Trimethacrylate
MMOFs	Microporous Metal–Organic Frameworks
MIHSs	Molecularly Imprinted Hollow Spheres
NIHSs	Nonimprinted Polymer Hollow Spheres
CL-20	Hexanitrohexaazaisowurtzitane
EGDMA	Ethylene Glycol Dimethacrylate
ACN	Acetonitrile
DMSO	Dimethyl Sulfoxide
KPS	Potassium Persulfate
APTES	3-Aminopropyltriethoxysilane
TEOS	Tetraethyl Orthosilicate
ACVA	4,4′-Azobis(4-cyanovaleric Acid)
DHEBA	N,N′-(1,2-Dihydroxyethylene)bisacrylamide
AAM	Acrylamide
FRET	Förster Resonance Energy Transfer
RDF	Radial Distribution Function
KBI	Kirkwood–Buff Integral
MICs	Molecularly Imprinted Colloidal Particles
NICs	Nonimprinted Colloidal Particles
NC	Nitrocellulose
FT-IR	Fourier Transform Infrared
SEM	Scanning Electron Microscopy
XRD	X-ray Diffraction
TGA	Thermogravimetric Analysis
RDX	1,3,5-trinitroperhydro-1,3,5-triazine
HMX	Octahydro-1,3,5,7-tetranitro-1,3,5,7-tetrazocine
TADB	Tetraacetyldibenzylhexaazaisowurtzitane
TATP	Triacetone Triperoxide
HMTD	Hexamethylene Triperoxide Diamine
Cz	Carbazole
TEAP	Tetraethylammonium Perchlorate
PBE	Peroxide-Based Explosive
PCz	Polycarbazole
SPME	Solid-Phase Microextraction
LPME	Liquid-Phase Microextraction
DLLME	Dispersive Liquid–Liquid Microextraction
AIBN	Azobisisobutyronitrile
LSV	Linear Sweep Voltammetry
CV	Cyclic Voltammetry
SWV	Square Wave Voltammetry
CA	Chronoamperometry
PM	Piezoelectric Microgravimetry
DFT	Density Functional Theory
SERS	Surface-Enhanced Raman Spectroscopy
CL	Chemiluminescence
SPR	Surface Plasmon Resonance
LSPR	Localized Surface Plasmon Resonance
PhC	Photonic Crystal
OWS	Optical Waveguide Spectroscopy
GNSs	Gold Nanostars
POF	Plastic Optical Fiber
MICA	Molecularly Imprinted Colloidal Array
QCM	Quartz Crystal Microbalance
MIPC	Molecularly Imprinted Photonic Crystal
NCs	Nanocrystals
3-NT	3-Nitrotoluene
NM	Nitromethane
PCL	Polycaprolactone
PL	Photoluminescence
GOx	Graphene Oxide
CNTs	Carbon Nanotubes
PETN	Pentaerythritol Tetranitrate
FAPA-MS	Flowing Atmospheric Pressure Afterglow Mass Spectrometry
RSD	Relative Standard Deviation
